# Unique sex chromosome translocations and evolutionary strata in two Sylvioidea songbird families

**DOI:** 10.1186/s12864-026-12861-1

**Published:** 2026-04-20

**Authors:** Thomas J. Brown, Simon J. Ellerstrand, Hanna Sigeman, Martim Melo, Max Lundberg, Bengt Hansson

**Affiliations:** 1 Department of Biology, Science for Life Laboratory, BECC – Biodiversity and Ecosystem services in a Changing Climate, Lund University, Lund, Sweden; 2 Ecology and Genetics Research Unit, University of Oulu, Oulu, Finland; 3 FitzPatrick Institute of African Ornithology, Department of Biological Sciences, University of Cape Town, Rondebosch, South Africa; 4 MHNC-UP, Natural History and Science Museum of the University of Porto, Porto, Portugal

**Keywords:** Sex chromosome evolution, Neo-sex chromosomes, Recombination suppression, Evolutionary strata, Birds

## Abstract

Avian genomes are typically stable, yet instances of chromosome fissions, fusions and translocations are known across various lineages. For example, multiple sex chromosome translocations have been described in the superfamily Sylvioidea. In this study, we examine the sex chromosomes in two Sylvioidea families, Nicatoridae and Cisticolidae, using whole-genome data. We found sex-linkage of chromosomes Z and 4A, consistent with findings in other Sylvioidea species. We also identify sex-linkage of parts of chromosome 4, marking a novel discovery for Nicatoridae and a previously documented feature in Cisticolidae. These families are non-sister taxa, and the size of the translocated region of chromosome 4 differs between them, supporting independent translocation events. We uncover variation in recombination suppression and evolutionary strata on chromosome 4 between families, as well as between two genera within Cisticolidae. This study reveals both recurrent and independent trajectories in sex chromosome evolution, deepening our understanding of complex genomes.

**Supplementary Information:**

The online version contains supplementary material available at 10.1186/s12864-026-12861-1.

## Introduction

Sexual reproduction is nearly universal to eukaryotic life, often resulting in the evolution of two separate sexes. However, the mechanisms by which individuals develop into males or females are highly diverse across taxa [1–4]. Closely related species, and even populations within species, can vary in their sex-determining mechanism [5–7]. Our understanding of the extent and drivers of this variation remains incomplete, even for well-studied taxa. In birds and mammals, sex is determined by heteromorphic sex chromosomes [4, 8]. The ZW (birds) and XY (mammals) chromosomes have evolved from different ancestral vertebrate autosomes more than 100 MYA, following the acquisition of a sex-determining gene [8, 9]. Subsequent accumulation of sexually antagonistic mutations (i.e., mutations beneficial to one sex and detrimental to the other) around the sex-determining gene is hypothesised to have favoured the evolution of recombination suppression across the sex chromosomes, which over time has resulted in severe degradation and gene loss on the non-recombining W/Y relative to their recombining Z/X counterparts [3, 10, 11].

Despite the sex chromosomes being highly conserved across the avian and mammalian phylogenies, there is an increasing awareness of the complex interplay of evolutionary processes that can produce highly variable sex chromosome structure and functionality across lineages [4, 8, 12, 13]. For example, the size of the pseudoautosomal regions (PARs)—the regions of the W/Y that continue to recombine with Z/X and thus avoid degradation—can vary considerably within clades, resulting in a spectrum of sex chromosome dimorphisms [12, 14, 15]. Furthermore, autosomes or sections of autosomes can fuse or be translocated to sex chromosomes to create “neo-sex chromosomes”. Neo-sex chromosomes represent a recurrent phenomenon, having been reported in a range of diverse taxa [16–18]. As is the case for ancestral sex chromosomes, neo-sex chromosomes can exhibit signatures of degeneration in the absence of recombination; however, their more recent sex-linkage implies that the degenerative process is at an earlier stage than ancestral sex chromosomes [11, 16, 19]. The generation of new sex-linked loci can play important roles in sex-specific evolution, such as resolving sexual antagonism [20–22] and driving speciation, whereby the new sex chromosome karyotype becomes reproductively isolated and contributes to local adaptation [17, 23, 24].

Our understanding of neo-sex chromosomes is limited in part by incomplete knowledge of interspecific variation in sex chromosome systems. However, recently developed bioinformatic methods now make it possible to characterise sex chromosomes in a range of non-model species [21, 25, 26]. Using these methods, recent studies have uncovered considerable variation in the origin, size and age of newly sex-linked chromosomal regions within lineages, suggesting a high turnover of neo-sex chromosomes [18, 27]. However, genome-wide comparisons of closely related species are needed to fully understand the occurrence of independently evolved neo-sex chromosomes [8].

One of the most comprehensively studied avian neo-sex chromosome systems [11, 19, 27–30] belongs to the songbird superfamily Sylvioidea (*sensu* Alström et al. [31]. and Fregin et al. [32]). Sylvioidea includes over 1,200 species across approximately 22 families that diverged from other passerine lineages around 25 MYA [31–33]. Comparative genomic analyses have revealed enlarged and complex sex chromosomes formed by multiple independent translocation events. In all Sylvioidea species studied to date, a region of a chromosome that is called 4A in *Taeniopygia guttata* (Zebra finch) has translocated to the ancestral Z and W chromosomes [19, 27–29, 34]. In addition to this phylogenetically basal Z–4A fusion, translocations of parts of Chrs 3, 4, 5 and 8 to the Z–4A neo-sex chromosome (and possibly to W–4A) have been discovered in four Sylvioidea families; Alaudidae (Chrs 3 and 5), Cisticolidae (Chr 4), Panuridae (Chr 3) and Macrosphenidae (Chr 8) [27, 30, 35]. Further evidence suggests that the translocated regions can have different evolutionary trajectories between families. For example, a part of the sex-linked Chr 4A still recombines in Macrosphenidae (*Sylvietta brachyura*) females (ZW), while it has ceased to recombine in all other Sylvioidea species studied to date [27, 30]. Moreover, the extent of W degeneration on homologous sex-linked regions varies between lineages [11].

Here, we expand knowledge of avian neo-sex chromosome diversity by searching for sex-linked chromosomal regions in a previously unstudied Sylvioidea species. We conduct whole-genome analyses of three species representing two Sylvioidea families—Nicatoridae and Cisticolidae—and two Cisticolidae genera (*Camaroptera* and *Cisticola*). Specifically, we examine the previously unstudied *Nicator vireo* (Yellow-throated nicator; Nicatoridae) and compare the neo-sex chromosomes of the previously studied *Camaroptera brevicaudata* (Grey-backed camaroptera; Cisticolidae) [11] and *Cisticola juncidis* (Zitting cisticola; Cisticolidae) [27]. We assess sex-linkage across the genome using two metrics derived from mapped whole-genome sequencing reads: differences between the sexes in sequencing depth and heterozygosity. These metrics detect sex chromosome regions with varying degrees of W degeneration [21, 26], reflecting the timing of recombination suppression between Z and W chromosomes [11, 36]. Older regions with highly degraded W chromosomes typically show male-biased sequencing depth and heterozygosity (few or no W reads map to the Z chromosome reference), whereas newer regions show female-biased heterozygosity and weak or no male-biased sequencing depth (many W reads, partially differentiated from Z, map to the Z reference) [21, 26]. By visualizing these metrics across a reference genome, we identify sex-linked regions and evolutionary strata—chromosome regions where Z–W recombination ceased at different time points [21, 26] (see also [36]). We discover that part of Chr 4 has been translocated to Z in *N. vireo*, and that this translocation is distinct from the Chr 4 translocation in Cisticolidae, implying independent neo-sex chromosome evolution in these two Sylvioidea families. Additionally, we uncover variation in the timing of recombination suppression and evolutionary strata across the sex-linked region of Chr 4 between families and between the two studied Cisticolidae genera.

## Methods

DNA extracted from blood samples of one female and one male *Nicator vireo* (Nicatoridae) and one female and one male *Cisticola juncidis* (Cisticolidae). These samples were subjected to paired-end sequencing (2 × 150 bp) on the Illumina NovaSeq 6000 platform. Library preparation and sequencing were performed at SciLifeLab (Uppsala, Sweden). Illumina paired-end reads (2 × 150 bp) for one female and one male *Camaroptera brevicaudata* (Cisticolidae) and an additional female and male *Cisticola juncidis* (Cisticolidae) were retrieved from NCBI (original sources: [11, 27]). Two species, *N. vireo* and *Cam. brevicaudata*, have a sub-Saharan African distribution, whereas *Cis. juncidis* occurs across Africa and Eurasia [37, 38]. Sample details and NCBI accession numbers are provided in Table S1.

Raw sequences were trimmed for quality and adapters with *Trimmomatic v.0.36* [39]. Bases with a phred score < 20 were trimmed from the leading and trailing ends in averages of 4 bases in sliding windows, and reads < 100 bp were discarded (ILLUMINACLIP: TruSeq3-PE-2.fa:2:30:10:1:TRUE LEADING:20 TRAILING:20 SLIDINGWINDOW:4:20 MINLEN:100). Only reads with both pairs retained were used for further downstream analyses. We built *de novo* scaffold-level genome assemblies using trimmed paired reads of a male sample of each species (Table S2), with *SPAdes v.3.15.3* [40], specifying six kmer lengths (21, 33, 55, 77, 99, 127). These short-read assemblies are highly fragmented (assembly statistics in Table S2), but nonetheless suitable for species-specific read mapping. As males, the homogametic sex, were used, W-linked scaffolds are not included in the assemblies.

Next, we employed *FindZX—*a bioinformatic pipeline for sex chromosome identification using short-read data [26]—to detect sex-linked regions in the genomes of the three species. *FindZX* is openly available at GitHub (https://github.com/hsigeman/findZX) and implements commonly used software, including *BWA-MEM* [41, 42] for read alignment, *SAMtools* [43] for filtering aligned read, and *Platypus* [44] for variant calling. In brief, we aligned the trimmed read-pairs of the female and male samples of each species (one sample per sex for *N. vireo* and *Cam. brevicaudata*; two samples per sex for *Cis. juncidis*) to their scaffold-level assemblies, filtered the aligned reads (low quality alignments, secondary alignments and read duplicates were removed), performed variant calling, and calculated the difference between sexes (heterogametic female - homogametic male) in (i) standardized sequencing depth (utilizing three alignment mismatch criteria; no filtering, ≤ 2 mismatches, and 0 mismatches), and (ii) % heterozygous sites, in 5 kb windows across scaffolds. Using the *FindZX* option *synteny-species reference genome* [26], we assigned chromosome coordinates to each 5 kb window of the species-specific scaffold-level assemblies based on alignment to the *Parus major* (Great tit) reference genome (Parus_major1.1, GCA_001522545.3; Table S3) [45], a non-Sylvioidea species in family Paridae. This enabled calculation and visualization of the female-to-male difference in sequencing depth and heterozygosity across entire chromosomes. We chose *P. major* as a reference species as it is more closely related to Sylvioidea than *T. guttata*, and because the Z chromosome of *T. guttata* shows substantial rearrangements to other songbirds [19]. *FindZX* outputs the difference between the heterogametic and homogametic sex in % heterozygous sites and sequencing depth in 50 kb, 100 kb and 1 Mb window sizes (mean values of 5 kb windows) across the reference genome for the three mismatch criteria. The *FindZX*-output for (i) female-to-male difference in % heterozygous sites and standardized sequencing depth across all chromosomes in 100 kb windows is listed in Table S4 and visualised in Figure S1, and (ii) sex-specific % heterozygous sites and standardized sequencing depth across all chromosomes in 1 Mb windows is visualised in Figure S2.

**Fig. 1:**
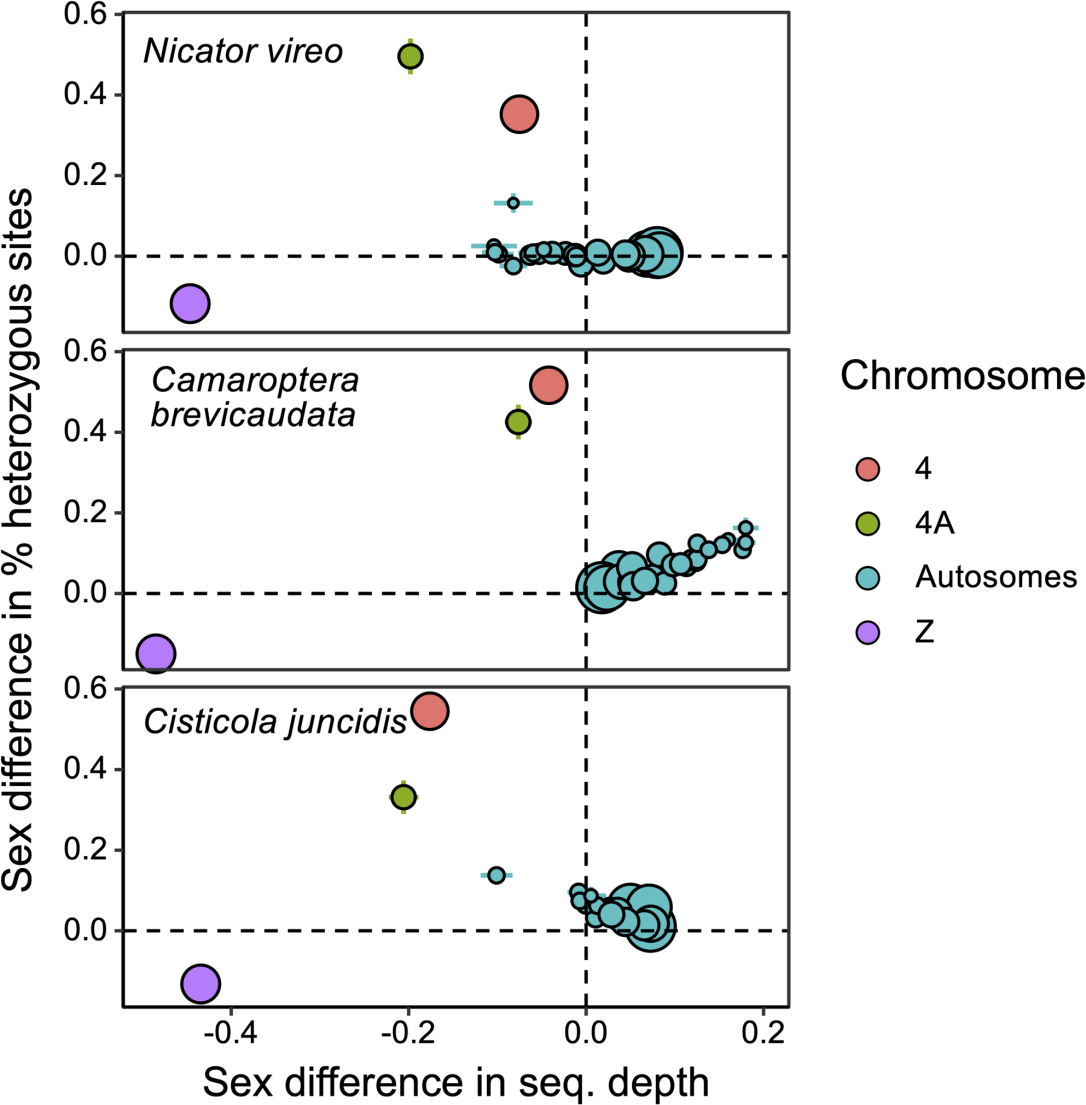
Sex difference (female-male) in sequencing depth and % heterozygous sites per chromosome in *Nicator vireo* (Nicatoridae), and *Camaroptera brevicaudata* and *Cisticola juncidis* (Cisticolidae). Mean ± s.e. values based on 100 kb windows are shown for each chromosome. Dot sizes correspond to chromosome lengths. Sex-linked chromosomes (Z, 4A and 4) are highlighted in distinct colours from autosomes. The identification of Chr 4 as sex-linked in *N. vireo* represents a novel finding in this study

**Fig. 2:**
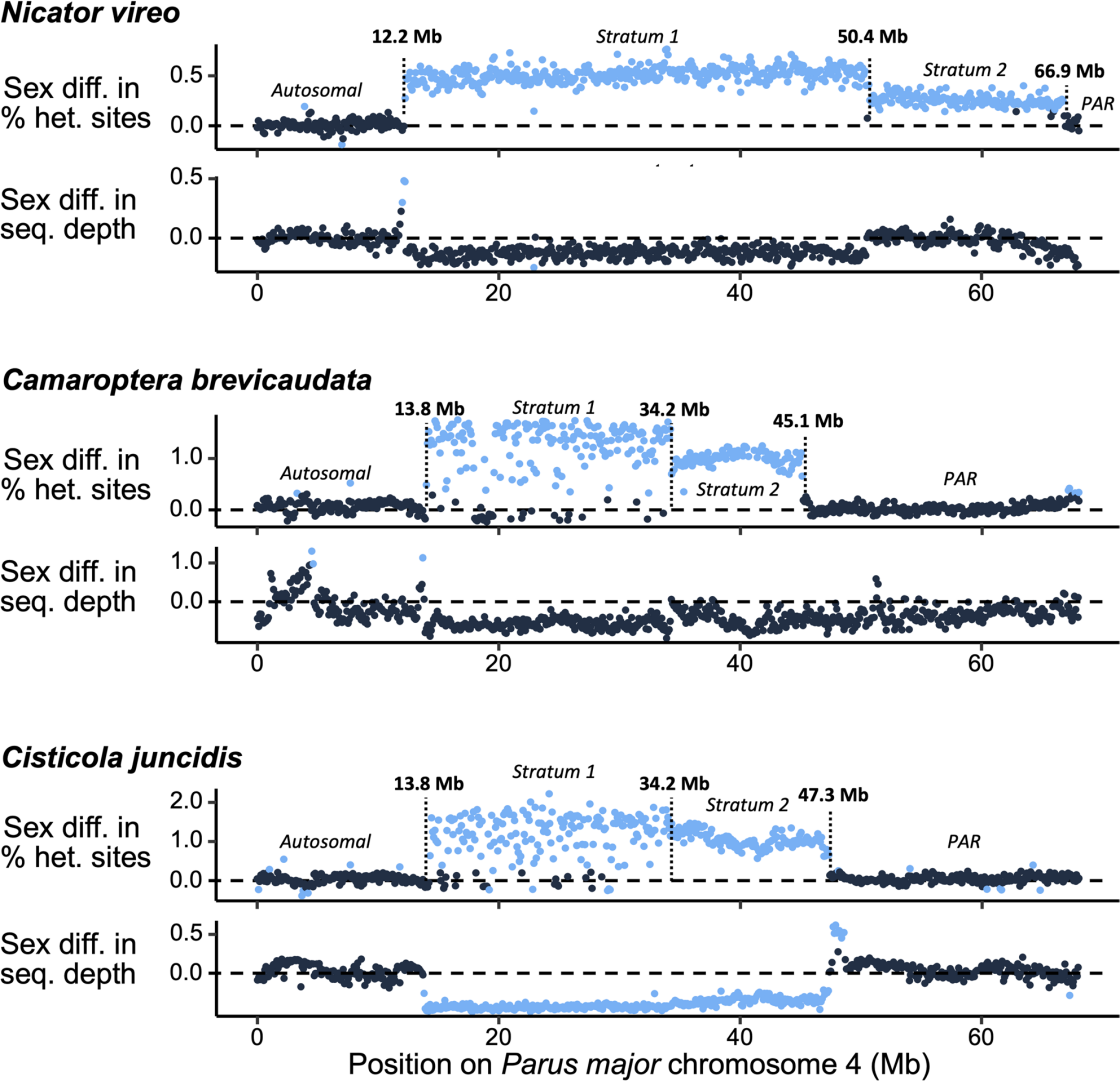
Sex difference (female-male) in (i) % heterozygous sites and (ii) sequencing depth across *Parus major* chromosome 4 in *Nicator vireo*, *Camaroptera brevicaudata* and *Cisticola juncidis*. Each dot represents a 100 kb window, and values deviating from the genome-wide pattern are given in blue. Indicated are start and end positions of strata, PAR and autosomal region

The output from *FindZX* was further analysed in *R v3.6.1* [46]. For each chromosome, mean and standard error for sex difference in standardized sequencing depth and % heterozygous sites were calculated based on data in 100 kb windows (Fig. 1). For sequencing depth, we used data from the ≤ 2 mismatches filter for *N. vireo* and *Cis. juncidis*, and unfiltered data for *Cam. brevicaudata*, as these settings provided the most informative broad-scale patterns (see results for all three mismatch criteria in Figure S1). To visualize the sex-linked pattern across Chr 4 (the novel finding in this study), we highlighted windows that strongly deviated from the genome-wide (autosomal) pattern by calculating species-specific outlier thresholds for each metric (sex differences in sequencing depth and heterozygosity; Fig. 2). Outliers were identified by calculating the interquartile range (IQR) and first and third quartiles (1Q and 3Q) across all chromosomes for each species separately. Any values lower than 1Q-IQR*1.5 or higher than 3Q + IQR*1.5 was categorized as an outlier (i.e., deviating from the genome-wide pattern).

Finally, we downloaded the available genome assemblies of *N. chloris* (Western nicator; no assembly was available for *N. vireo*) and *Cis. juncidis* (NCBI accession numbers and assembly statistics are given in Table S3). To search for evidence supporting for the fusion between Chr 4 and Z–4A (i.e., single scaffolds showing homology to both Chr 4 and Chr Z or 4A), and the PAR-boundary (i.e., single scaffolds spanning the transition between non-recombining–recombining regions on Chr 4), scaffolds from each assembly were aligned to the chromosome-level reference assembly of *P. major* using *SatsumaSynteny 2.0* [47] (Table S5).

## Results

Sequencing depth and heterozygosity differed strongly between the sexes on three chromosomes (Chr Z, 4A and 4) in *N. vireo*, *Cam. brevicaudata* and *Cis. juncidis* (Fig. 1, S1, S2; Table S4). In all three species, females (ZW) showed substantially reduced sequencing depth and lower heterozygosity on Chr Z compared with males (ZZ), consistent with expectations that heterogametic females carry a single Chr Z sequence following extensive W degeneration. As expected under such hemizygous conditions, females exhibited near-zero heterozygosity on Chr Z (Figure S2). On Chr 4A (of which part is sex-linked in all Sylvioidea species studied so far) the females showed moderately lower sequencing depth and substantially elevated heterozygosity compared to the males. These genomic signatures are consistent with the expectation for a neo-sex chromosome region, where degradation on the W-copy is less severe than for an ancestral W. Interestingly, Chr 4 (previously identified as sex-linked in Cisticolidae) showed a similar signature of sex-linkage as for Chr 4 A in all three species, which thus represents a novel finding of a neo-sex chromosome region in *N. vireo*. In *N. vireo* and *Cam. brevicaudata*, several micro-chromosomes showed some degree of sex-bias in sequencing depth, with a few also exhibiting slight deviations in heterozygosity (Fig. 1). However, due to their small size (and thus limited data) and minor sex difference in heterozygosity, we do not infer any of them as being sex-linked. Other chromosomes (including Chr 3, 5 and 8 known to be sex-linked in other Sylvioidea species; [30, 35]) exhibited weak or no sex-bias in sequencing depth and heterozygosity (Fig. 1, S1; Table S4).

Regarding the three chromosomes with substantial sex-difference in sequencing depth and/or heterozygosity, Chr Z showed clear signs of sex-linkage across its entire length, and Chr 4A between positions 0–9.4 Mb, in all three species (Figure S1; Table S4;). In contrast, for Chr 4 the extent of the sex-linkage varied between species. In *N. vireo*, the sex-linked signal spanned positions 12.2–66.9 Mb, while in *Cam. brevicaudata* and *Cis. juncidis*, the signal spanned positions 13.8–45.1 Mb and 13.8–47.3 Mb, respectively (Fig. 2; Table S4). Furthermore, in *N. vireo* the sex differentiation was more pronounced in region 12.2–50.4 Mb than in region 50.4–66.9 Mb (Fig. 2; Table S4), indicating two different evolutionary strata with different timing of recombination suppression—stratum 1 (12.2–50.4 Mb) being older than stratum 2 (50.4–66.9 Mb). Similar patterns were observed for the two Cisticolidae species, albeit with different coordinates: stratum 1 between 13.8 and 34.2 Mb and stratum 2 between 34.2 and 45.1 Mb in *Cam. brevicaudata*, and stratum 1 between 13.8 and 34.2 Mb and stratum 2 between 34.2 and 47.3 Mb in *Cis. juncidis* (Fig. 2; Table S4).

Analysis of *N. chloris* and *Cis. juncidis* scaffolds aligned to the *P. major* reference assembly indicates that position 66.9 Mb in *N. vireo* and position 47.3 Mb in *Cis. juncidis* mark the PAR-boundary on Chr 4—the transition between the non-recombining and recombining region of the neo-sex chromosome—as each position is spanned by a single scaffold (a 101 kb scaffold for *N. chloris* and a 1.04 Mb scaffold for *Cis. juncidis*; Table S5). However, we found no direct evidence that the other recombination/non-recombination position represents the fusion site in either species, as scaffolds flanking these positions on Chr 4 showed no homology to Chr Z or 4 A (Table S5).

## Discussion

Using whole-genome analyses in *Nicator vireo* (family Nicatoridae), we confirmed sex-linkage of parts of Chr 4 A in yet another Sylvioidea family (cf. [27–29, 48]) and identified a previously unreported translocation involving a large region of Chr 4. Notably, Chr 4 is also sex-linked in Cisticolidae [27] (this study)—a non-sister taxon to Nicatoridae [32, 33]—implying that Chr 4 has undergone at least two independent translocation within Sylvioidea, in the lineages leading to Nicatoridae and Cisticolidae, respectively. In family Macrosphenidae, which phylogenetically falls between Nicatoridae and Cisticolidae [33], Chr 4 is not sex-linked [30]. Nicatoridae and Cisticolidae represent deep branches within the Sylvioidea phylogeny, making precise dating of these translocation events challenging. A published dated phylogeny for Passeriformes [33] indicate that Nicatoridae diverged from other families approximately 24 MYA, providing a maximum age of the translocation event in *N. vireo*. Based on the same source [33], together with a more detailed phylogeny of Cisticolidae [32], the translocation in Cisticolidae should have occurred between ≈ 23 and ≈ 11 MYA—after the formation of Cisticolidae but before the divergence of *Cisticola* and *Camaroptera*. The evolutionary drivers of these translocations remain uncertain. However, genes with sex-related functional annotations, i.e., gene categories of particular relevance for processes promoting sex-linkage and recombination suppression [3, 4], are present in the translocated regions of both Chr 4 and 4A (a full list of protein coding genes on Chr 4 and 4A in *Parus major*, and their GO terms, are provided in Table S6; downloaded from https://www.ensembl.org/index.html, Ensembl v.115).

The micro-chromosomes of *N. vireo* and *Cam. brevicaudata* exhibited some sex-bias in sequencing depth, with a few also exhibiting slight variation in heterozygosity (Fig. 1). However, the direction of the patterns differs between species, complicating interpretation. In *Cam. brevicaudata*, smaller chromosomes displayed slightly higher depth and heterozygosity in females—an unexpected pattern for sex-linked regions, possibly reflecting W-derived reads mapping to these chromosomes for unknown reasons. In contrast, *N. vireo* showed somewhat lower female coverage on smaller chromosomes, with one also displaying a slight increase in heterozygosity. A similar pattern was observed for one micro-chromosome in *Cis. juncidis*. Given the small size (and thus limited data) of these chromosomes and the moderate sex-bias in depth and heterozygosity, we do not infer any of them as being sex-linked. Moreover, these differences are minor compared to the strong signals associated with the recently translocated regions of Chr 4A and Chr 4.

**Fig. 3:**
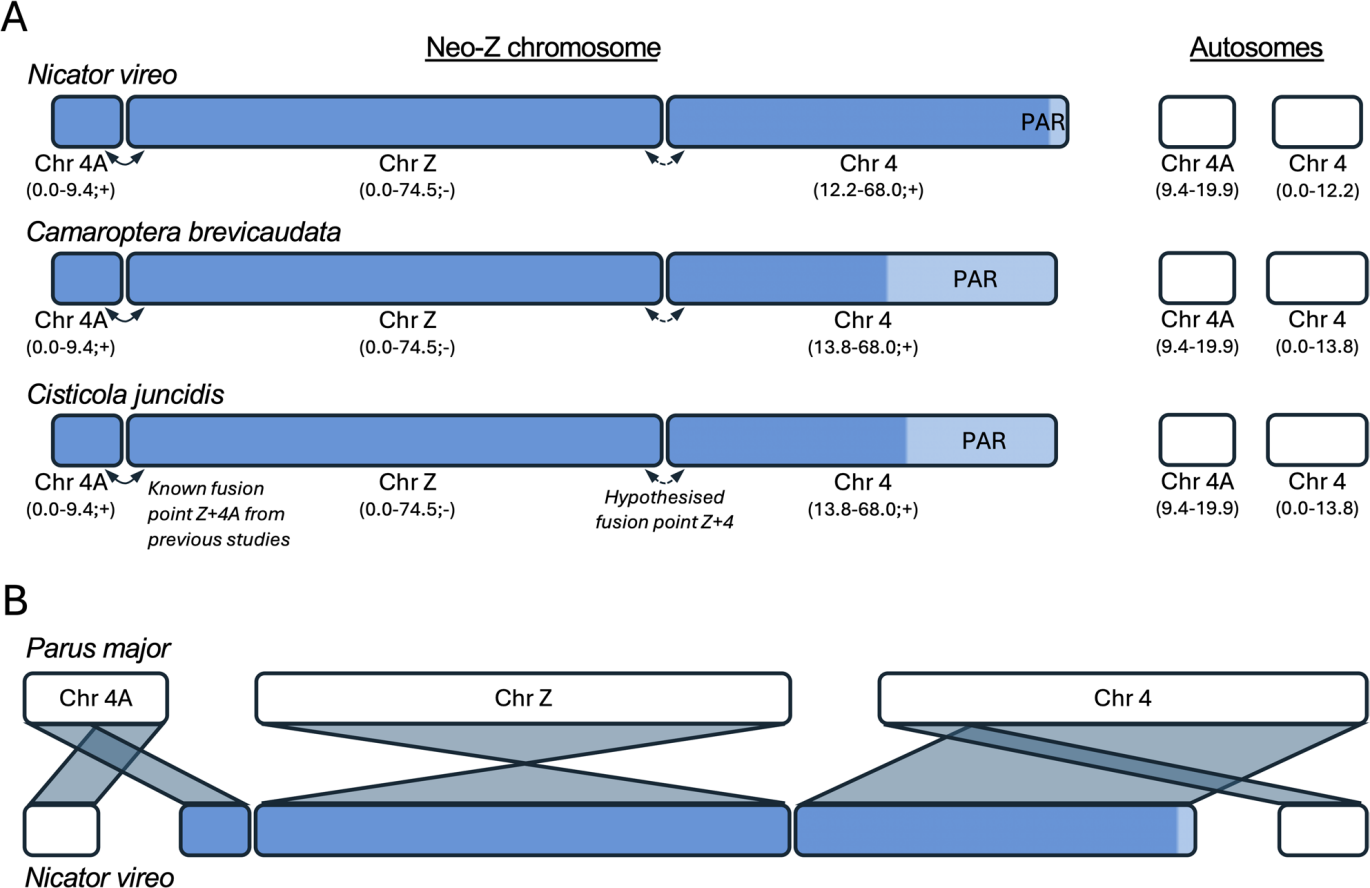
**A** Putative structure of the neo-Z chromosome of *Nicator vireo*, *Camaroptera brevicaudata* and *Cisticola juncidis*. Indicated are non-recombining (dark blue) and PAR (light blue) regions, and start and end positions (and direction) of homologous chromosomes of *Parus major* (in parentheses). Also indicated are autosomal parts of Chrs 4A and 4. **B** Inferred synteny across Chrs Z, 4 and 4A between *Parus major* and *Nicator vireo*

In both Nicatoridae and Cisticolidae, the sex-linked, non-recombining region on Chr 4 is flanked by recombining regions with no sex bias on both sides (Fig. 2). This suggests that Chr 4 was split before the translocation, with one flanking recombining region representing an independently segregating autosome. Similar patterns occur for Chr 4A in Sylvioidea [27, 49, 50] and Chr 8 in Macrosphenidae [30]. In *T. guttata*, the centromere is located at the beginning of Chr 4, and because an independently segregating chromosome requires a functional centromere, this suggests that the first recombining region of Chr 4 remains autosomal in Nicatoridae (0–12.2 Mb) and Cisticolidae (0–13.8 Mb). Therefore, the recombining–non-recombining boundary at position 12.2 Mb in *N. vireo* and 13.8 Mb in Cisticolidae most likely marks the approximate position of the fission/fusion points. This hypothesis is indirectly supported by single scaffolds spanning the opposite boundary—the PAR-boundary—at position 66.9 Mb in *N. vireo* and 47.3 Mb in *Cis. juncidis* (based on scaffolds aligning to the *P. major* reference; Table S5). Thus, we conclude that the fusion between Chr 4 and the rest of the Z chromosome (ancestral Chr Z plus the sex-linked region of Chr 4A) occurred at position 12.2 Mb in *N. vireo* and at 13.8 Mb in Cisticolidae. These contrasting fusion points provide further support for independent translocations of Chr 4 in Nicatoridae and Cisticolidae. In contrast, the shared recombining–non-recombining boundary at position 13.8 Mb in *Cam. brevicaudata* and *Cis. juncidis* suggests that a single translocation occurred early in the Cisticolidae lineage. Notice that all indicated positions are approximate and refer to the positions in the *Parus major* genome assembly. Schematic illustrations of the putative neo-Z structure of Nicatoridae and Cisticolidae and inferred synteny with *P. major* are shown in Fig. 3A and B.

In *Cam. brevicaudata*, the PAR-boundary is located at position 45.1 Mb, which differs from that of *Cis. juncidis* (47.3 Mb). This results in PAR lengths of 22.9 Mb (45.1–68.0 Mb) in *Cam. brevicaudata* and 20.7 Mb (47.3–68.0 Mb) in *Cis. juncidis*, both substantially larger than the PAR of *N. vireo* (1.1 Mb; 66.9–68.0 Mb) and the ancestral PAR on the Z chromosome in other passerines (≈ 0.5 Mb) [12, 50]. Interestingly, the male-to-female sequencing depth and heterozygosity patterns indicate two evolutionary strata on the sex-linked region of Chr 4 in each species. Stratum 1 appears shared between *Cam. brevicaudata* and *Cis. juncidis*, spanning the same region (13.8–34.2 Mb), suggesting it formed prior to the split of these genera. In contrast, stratum 2 differs in size (34.2–45.1 Mb in *Cam. brevicaudata*; 34.2–47.3 Mb in *Cis. juncidis*), indicating independent evolutionary events. In *N. vireo*, stratum 1 spans 12.2–50.4 Mb and stratum 2 50.4–66.9 Mb. The chromosomal order of strata and PAR (stratum 1 – stratum 2 – PAR) fits the expected progression of recombination suppression from the fusion point toward the chromosome end in all three species.

The discovery of a unique sex chromosome translocation in Nicatoridae adds further complexity to the evolutionary history of neo-sex chromosome system in Sylvioidea. To date, at least six translocation events to the Z chromosome, involving parts of five chromosomes (Chr 3, 4[×2], 4A, 5 and 8), have been identified as contributing to the Z chromosome structure [27, 30, 34, 35] (this study). The remarkable diversity of sex chromosome configurations in Sylvioidea challenges the long-standing notion of sex chromosome stability in birds [51–53]. Moreover, the variation in breakpoint locations, recombination suppression, evolutionary strata and PAR size in Nicatoridae and Cisticolidae provides new insights into the dynamic nature of avian sex chromosome evolution.

## Supplementary Information

Supplementary Material 1.

Supplementary Material 2.

## Data Availability

Illumina sequence reads have been deposited in the NCBI Sequence Read Archive (Bioproject PRJNA578893; Table S1).

## References

[1] Ohno S. Sex chromosomes and sex-linked genes. Berlin, Germany: Springer; 1967.

[2] Bull JJ. Evolution of sex determining mechanisms. Menlo Park, CA: Benjamin/Cummings Publishing Company; 1983.

[3] Bachtrog D, Kirkpatrick M, Mank JE, McDaniel SF, Pires JC, Rice W, Valenzuela N. Are all sex chromosomes created equal? Trends Genet. 2011;27(9):350–7. 21962970 10.1016/j.tig.2011.05.005

[4] Zhu Z, Younas L, Zhou Q. Evolution and regulation of animal sex chromosomes. Nat Rev Genet. 2025;26:59–74. 10.1038/s41576-024-00757-3 39026082

[5] Chandler CH. Cryptic intraspecific variation in sex determination in *Caenorhabditis elegans* revealed by mutations. Heredity (Edinb). 2010;105(5):473–82. 20502478 10.1038/hdy.2010.62

[6] Pennell MW, Mank JE, Peichel CL. Transitions in sex determination and sex chromosomes across vertebrate species. Mol Ecol. 2018;27(19):3950–63. 29451715 10.1111/mec.14540 PMC6095824

[7] Pen I, Uller T, Feldmeyer B, Harts A, While GM, Wapstra E. Climate-driven population divergence in sex-determining systems. Nature. 2010;468(7322):436–8. 20981009 10.1038/nature09512

[8] Bachtrog D, Mank JE, Peichel CL, Kirkpatrick M, Otto SP, Ashman TL, Hahn MW, Kitano J, Mayrose I, Ming R, et al. Sex determination: why so many ways of doing it? PLoS Biol. 2014;12(7):e1001899. 24983465 10.1371/journal.pbio.1001899 PMC4077654

[9] Cortez D, Marin R, Toledo-Flores D, Froidevaux L, Liechti A, Waters PD, Grutzner F, Kaessmann H. Origins and functional evolution of Y chromosomes across mammals. Nature. 2014;508(7497):488–93. 24759410 10.1038/nature13151

[10] Xu L, Auer G, Peona V, Suh A, Deng Y, Feng S, Zhang G, Blom MPK, Christidis L, Prost S, et al. Dynamic evolutionary history and gene content of sex chromosomes across diverse songbirds. Nat Ecol Evol. 2019;3(5):834–44. 30936435 10.1038/s41559-019-0850-1

[11] Sigeman H, Downing PA, Zhang H, Hansson B. The rate of W chromosome degeneration across multiple avian neo-sex chromosomes. Sci Rep. 2024;14(1):16548. 39020011 10.1038/s41598-024-66470-7 PMC11255319

[12] Zhou Q, Zhang J, Bachtrog D, An N, Huang Q, Jarvis ED, Gilbert MT, Zhang G. Complex evolutionary trajectories of sex chromosomes across bird taxa. Science. 2014;346(6215):1246338. 25504727 10.1126/science.1246338 PMC6445272

[13] Darolti I, Wright AE, Sandkam BA, Morris J, Bloch NI, Farre M, Fuller RC, Bourne GR, Larkin DM, Breden F, et al. Extreme heterogeneity in sex chromosome differentiation and dosage compensation in livebearers. Proc Natl Acad Sci U S A. 2019;116(38):19031–6. 31484763 10.1073/pnas.1905298116 PMC6754558

[14] Vicoso B, Kaiser VB, Bachtrog D. Sex-biased gene expression at homomorphic sex chromosomes in emus and its implication for sex chromosome evolution. Proc Natl Acad Sci U S A. 2013;110(16):6453–8. 23547111 10.1073/pnas.1217027110 PMC3631621

[15] Xu L, Wa Sin SY, Grayson P, Edwards SV, Sackton TB. Evolutionary dynamics of sex chromosomes of Paleognathous birds. Genome Biol Evol. 2019;11(8):2376–90. 31329234 10.1093/gbe/evz154 PMC6735826

[16] Zhou Q, Wang J, Huang L, Nie W, Wang J, Liu Y, Zhao X, Yang F, Wang W. Neo-sex chromosomes in the black muntjac recapitulate incipient evolution of mammalian sex chromosomes. Genome Biol. 2008;9(6):R98. 18554412 10.1186/gb-2008-9-6-r98 PMC2481430

[17] Kitano J, Ross JA, Mori S, Kume M, Jones FC, Chan YF, Absher DM, Grimwood J, Schmutz J, Myers RM, et al. A role for a neo-sex chromosome in stickleback speciation. Nature. 2009;461(7267):1079–83. 19783981 10.1038/nature08441 PMC2776091

[18] Huang Z, De OFI, Liu J, Peona V, Gomes AJB, Cen W, Huang H, Zhang Y, Chen D, Xue T, et al. Recurrent chromosome reshuffling and the evolution of neo-sex chromosomes in parrots. Nat Commun. 2022;13(1):944. 35177601 10.1038/s41467-022-28585-1 PMC8854603

[19] Sigeman H, Strandh M, Proux-Wera E, Kutschera VE, Ponnikas S, Zhang H, Lundberg M, Soler L, Bunikis I, Tarka M, et al. Avian neo-sex chromosomes reveal dynamics of recombination suppression and W degeneration. Mol Biol Evol. 2021;38(12):5275–91. 34542640 10.1093/molbev/msab277 PMC8662655

[20] Zhou Q, Bachtrog D. Sex-specific adaptation drives early sex chromosome evolution in Drosophila. Science. 2012;337(6092):341–5. 22822149 10.1126/science.1225385 PMC4107656

[21] Palmer DH, Rogers TF, Dean R, Wright AE. How to identify sex chromosomes and their turnover. Mol Ecol. 2019;28(21):4709–24. 31538682 10.1111/mec.15245 PMC6900093

[22] Bachtrog D. The Y Chromosome as a Battleground for Intragenomic Conflict. Trends Genet. 2020;36(7):510–22. 32448494 10.1016/j.tig.2020.04.008 PMC8329999

[23] Bracewell RR, Bentz BJ, Sullivan BT, Good JM. Rapid neo-sex chromosome evolution and incipient speciation in a major forest pest. Nat Commun. 2017;8(1):1593. 29150608 10.1038/s41467-017-01761-4 PMC5693900

[24] Shakya SB, Wang-Claypool CY, Cicero C, Bowie RCK, Mason NA. Neo-sex chromosome evolution and phenotypic differentiation across an elevational gradient in horned larks (*Eremophila alpestris*). Mol Ecol. 2022;31(6):1783–99. 35048444 10.1111/mec.16357

[25] Muyle A, Kafer J, Zemp N, Mousset S, Picard F, Marais GA. SEX-DETector: a probabilistic approach to study sex chromosomes in non-model organisms. Genome Biol Evol. 2016;8(8):2530–43. 27492231 10.1093/gbe/evw172 PMC5010906

[26] Sigeman H, Sinclair B, Hansson B. FindZX: an automated pipeline for detecting and visualising sex chromosomes using whole-genome sequencing data. BMC Genom. 2022;23(1):328. 10.1186/s12864-022-08432-9 PMC9044604 35477344

[27] Sigeman H, Ponnikas S, Hansson B. Whole-genome analysis across 10 songbird families within Sylvioidea reveals a novel autosome-sex chromosome fusion. Biol Lett. 2020;16(4):20200082. 32315592 10.1098/rsbl.2020.0082 PMC7211462

[28] Pala I, Naurin S, Stervander M, Hasselquist D, Bensch S, Hansson B. Evidence of a neo-sex chromosome in birds. Heredity (Edinb). 2012;108(3):264–72. 21897438 10.1038/hdy.2011.70 PMC3282394

[29] Leroy T, Anselmetti Y, Tilak M-K, Bérard S, Csukonyi L, Gabrielli M, Scornavacca C, Milá B, Thébaud C, Nabholz B. A bird’s white-eye view on neo- sex chromosome evolution. Peer Community J. 2021;1:e63.

[30] Sigeman H, Zhang H, Ali Abed S, Hansson B. A novel neo-sex chromosome in *Sylvietta brachyura* (Macrosphenidae) adds to the extraordinary avian sex chromosome diversity among Sylvioidea songbirds. J Evol Biol. 2022;35:1797–805. 36156325 10.1111/jeb.14096 PMC10087220

[31] Alström P, Ericson PG, Olsson U, Sundberg P. Phylogeny and classification of the avian superfamily Sylvioidea. Mol Phylogenet Evol. 2006;38(2):381–97. 16054402 10.1016/j.ympev.2005.05.015

[32] Fregin S, Haase M, Olsson U, Alstrom P. New insights into family relationships within the avian superfamily Sylvioidea (Passeriformes) based on seven molecular markers. BMC Evol Biol. 2012;12:157. 22920688 10.1186/1471-2148-12-157 PMC3462691

[33] Oliveros CH, Field DJ, Ksepka DT, Barker FK, Aleixo A, Andersen MJ, Alstrom P, Benz BW, Braun EL, Braun MJ, et al. Earth history and the passerine superradiation. Proc Natl Acad Sci U S A. 2019;116(16):7916–25. 30936315 10.1073/pnas.1813206116 PMC6475423

[34] Dierickx EG, Sin SYW, van Veelen HPJ, Brooke MdL, Liu Y, Edwards SV, Martin SH. Genetic diversity, demographic history and neo-sex chromosomes in the critically endangered Raso lark. Proc Biol Sci. 2020;287(1922):20192613. 32126957 10.1098/rspb.2019.2613 PMC7126062

[35] Sigeman H, Ponnikas S, Chauhan P, Dierickx E, Brooke ML, Hansson B. Repeated sex chromosome evolution in vertebrates supported by expanded avian sex chromosomes. Proc Biol Sci. 2019;286(1916):20192051. 10.1098/rspb.2019.2051 PMC6939255 31771477

[36] Zhang H, Sigeman H, Hansson B. Assessment of phylogenetic approaches to study the timing of recombination cessation on sex chromosomes. J Evol Biol. 2022;35:1721–33. 35895083 10.1111/jeb.14068 PMC10086819

[37] Winkler DW, Billerman SM, Lovette IJ. Nicators (Nicatoridae), version 1.0. Ithaca. NY, USA: Cornell Lab of Ornithology; 2020.

[38] Winkler DW, Billerman SM, Lovette IJ. Cisticolas and allies (Cisticolidae), version 1.0. Ithaca. NY, USA: Cornell Lab of Ornithology; 2020.

[39] Bolger AM, Lohse M, Usadel B. Trimmomatic: a flexible trimmer for Illumina sequence data. Bioinformatics. 2014;30(15):2114–20. 24695404 10.1093/bioinformatics/btu170 PMC4103590

[40] Prjibelski A, Antipov D, Meleshko D, Lapidus A, Korobeynikov A. Using SPAdes de novo assembler. Curr protocols Bioinf / editoral board Andreas D Baxevanis [et al]. 2020;70(1):e102. 10.1002/cpbi.102 32559359

[41] Li H, Durbin R. Fast and accurate long-read alignment with Burrows-Wheeler transform. Bioinformatics. 2010;26(5):589–95. 20080505 10.1093/bioinformatics/btp698 PMC2828108

[42] Li H. Aligning sequence reads, clone sequences and assembly contigs with BWA-MEM. *arXiv preprint* 2013:arXiv:1303.3997.

[43] Li H, Handsaker B, Wysoker A, Fennell T, Ruan J, Homer N, Marth G, Abecasis G, Durbin R. Genome Project Data Processing S: The sequence alignment/map format and SAMtools. Bioinformatics. 2009;25(16):2078–9. 19505943 10.1093/bioinformatics/btp352 PMC2723002

[44] Rimmer A, Phan H, Mathieson I, Iqbal Z, Twigg SRF, Consortium WGS, Wilkie AOM, McVean G, Lunter G. Integrating mapping-, assembly- and haplotype-based approaches for calling variants in clinical sequencing applications. Nat Genet. 2014;46(8):912–8. 25017105 10.1038/ng.3036 PMC4753679

[45] Laine VN, Gossmann TI, Schachtschneider KM, Garroway CJ, Madsen O, Verhoeven KJ, de Jager V, Megens HJ, Warren WC, Minx P, et al. Evolutionary signals of selection on cognition from the great tit genome and methylome. Nat Commun. 2016;7:10474. 26805030 10.1038/ncomms10474 PMC4737754

[46] Team RC. R: a language and environment for statistical computing. Vienna (Austria): R Foundation for Statistical Computing (http://www.R-project.org/); 2023.

[47] Grabherr MG, Russell P, Meyer M, Mauceli E, Alfoldi J, Di Palma F, Lindblad-Toh K. Genome-wide synteny through highly sensitive sequence alignment: Satsuma. Bioinformatics. 2010;26(9):1145–51. 20208069 10.1093/bioinformatics/btq102 PMC2859124

[48] Sigeman H, Ponnikas S, Videvall E, Zhang H, Chauhan P, Naurin S, Hansson B. Insights into avian incomplete dosage compensation: sex-biased gene expression coevolves with sex chromosome degeneration in the common whitethroat. Genes. 2018;9(8):373. 30049999 10.3390/genes9080373 PMC6116046

[49] Sigeman H, Hansson B. Evolutionary dynamics of enlarged sex chromosomes and novel pseudoautosomal regions in Sylvioidea songbirds. In: *Evolution of sex chromosomes in Sylvioidea songbirds.* Edited by Sigeman H: PhD Thesis, Lund University; 2021: 163–185.

[50] Ponnikas S, Sigeman H, Lundberg M, Hansson B. Extreme variation in recombination rate and genetic diversity along the Sylvioidea neo-sex chromosome. Mol Ecol. 2022;31(13):3566–83. 35578784 10.1111/mec.16532 PMC9327509

[51] Nanda I, Shan Z, Schartl M, Burt DW, Koehler M, Nothwang H, Grutzner F, Paton IR, Windsor D, Dunn I, et al. 300 million years of conserved synteny between chicken Z and human chromosome 9. Nat Genet. 1999;21(3):258–9. 10080173 10.1038/6769

[52] Nanda I, Schlegelmilch K, Haaf T, Schartl M, Schmid M. Synteny conservation of the Z chromosome in 14 avian species (11 families) supports a role for Z dosage in avian sex determination. Cytogenet Genome Res. 2008;122(2):150–6. 19096210 10.1159/000163092

[53] Ellegren H. Evolutionary stasis: the stable chromosomes of birds. Trends Ecol Evol. 2010;25(5):283–91. 20363047 10.1016/j.tree.2009.12.004

