# Supplementary material for: Unique sex chromosome translocations and evolutionary strata in two Sylvioidea songbird families
Source: BMC Genomics. 2026 Apr 20;27:401. doi: 10.1186/s12864-026-12861-1 (PMC13097842; doi:10.1186/s12864-026-12861-1)
Supplement: Supplementary file 1 — Supplementary Material 1. [file 12864_2026_12861_MOESM1_ESM.docx]

Supplementary Figures S1 and S2

Title: Unique sex chromosome translocations and evolutionary strata in two Sylvioidea songbird families

Authors: Thomas Brown, Simon Jacobsen Ellerstrand, Hanna Sigeman, Martim Melo, Max Lundberg, Bengt Hansson

# Figure S1

A) *Nicator vireo*


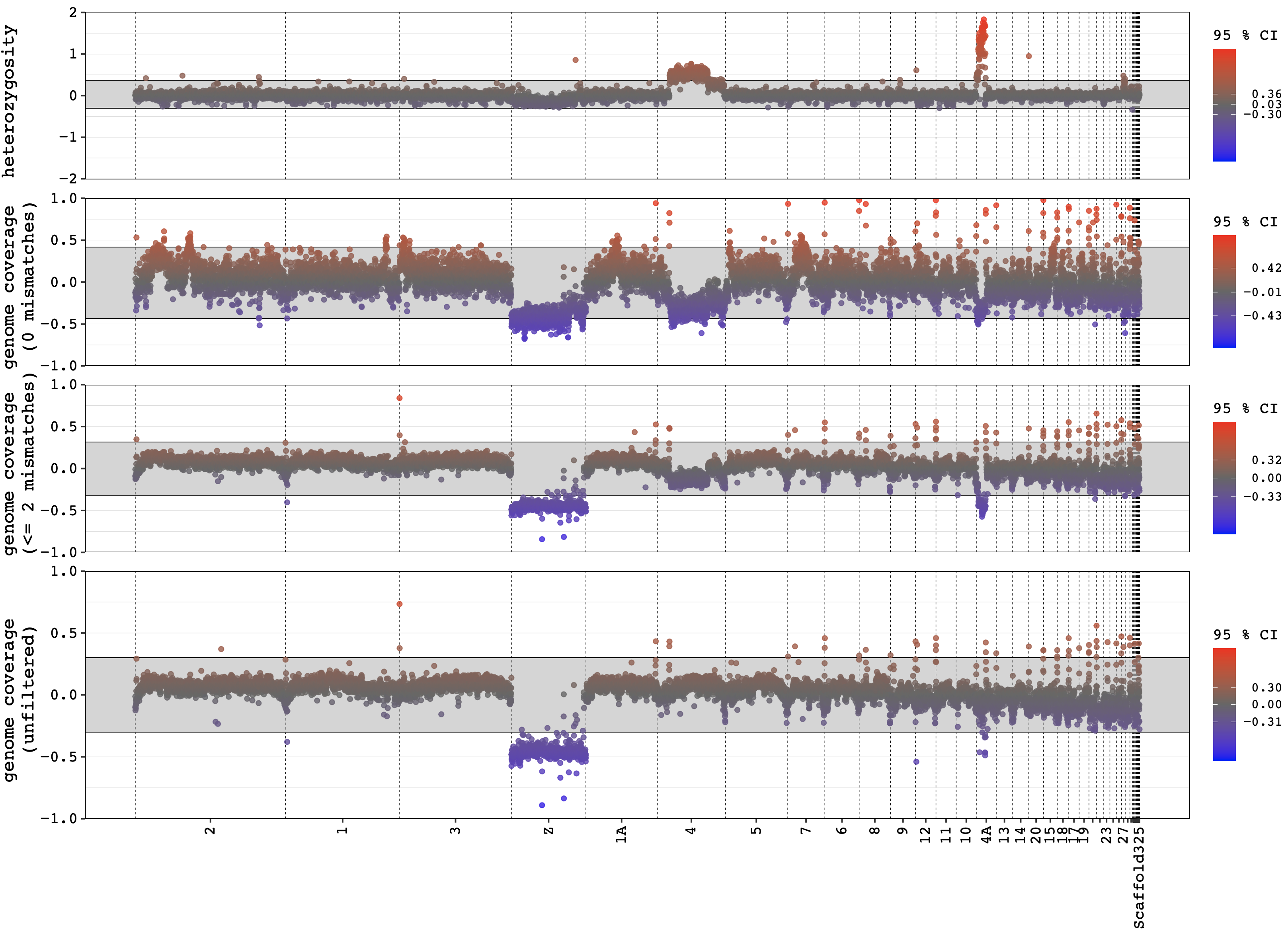


B) *Camaroptera brevicaudata*


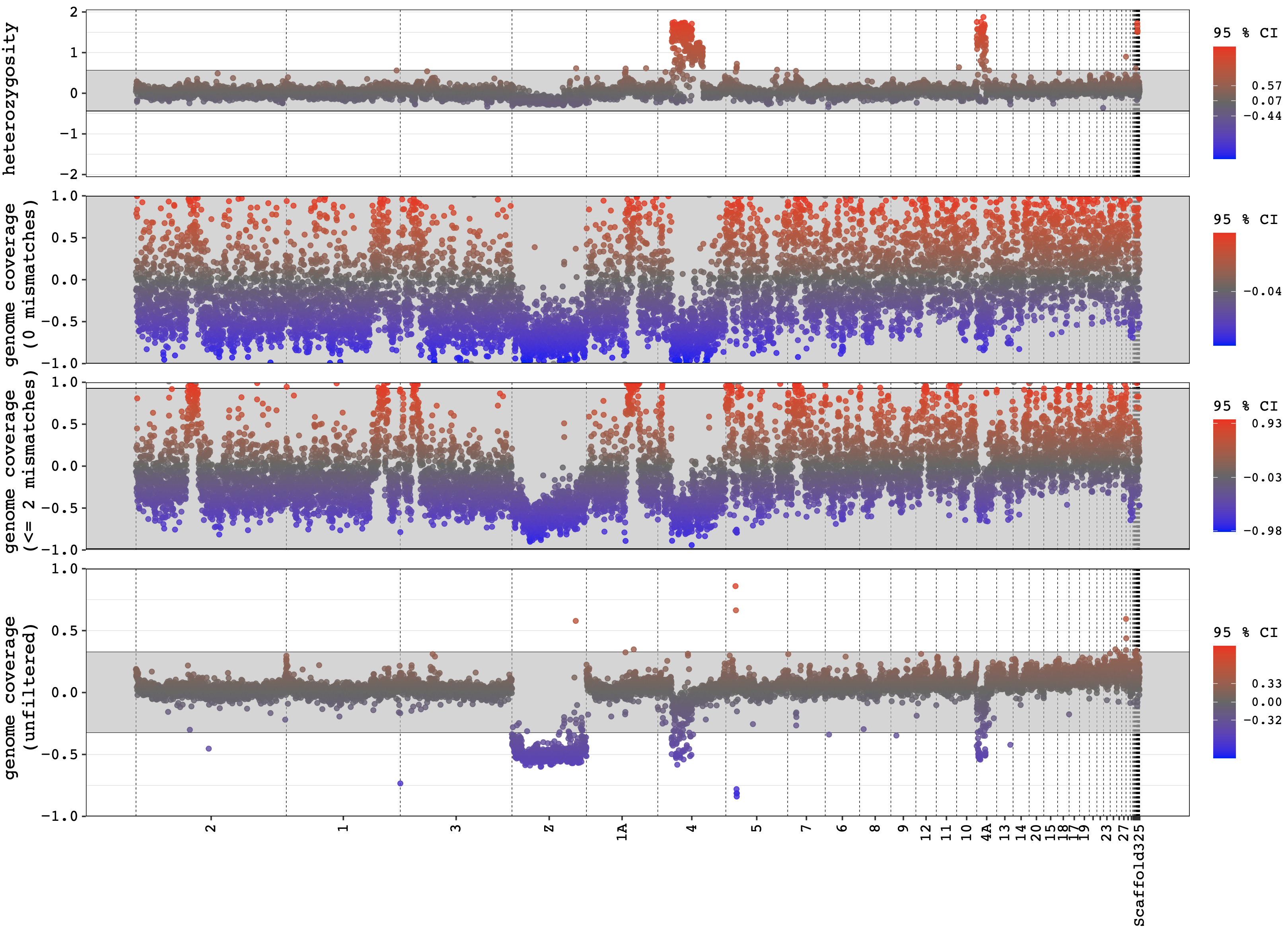


C) *Cisticola juncidis*


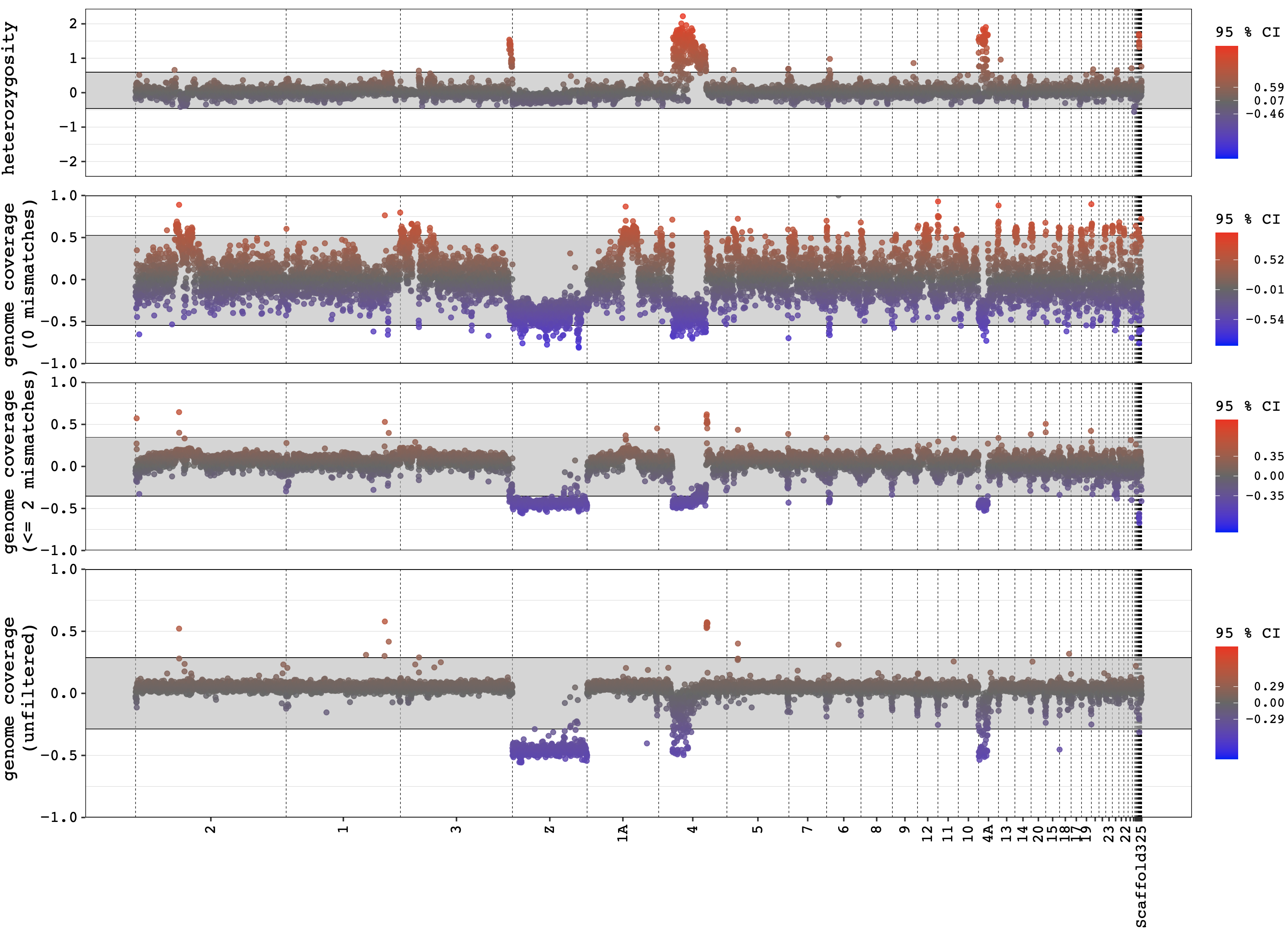


**Figure S1.** Female-to-male difference in heterozygosity and sequencing depth (genome coverage) across *Parus major* chromosomes (numbered accordingly) in 100 kb windows for (A) *Nicator vireo*, (B) *Camaroptera brevicaudatus* and (C) *Cisticola juncidis*. Sequencing depth is based on data of three different read mismatch criteria (see main text for details).

# Figure S2

A) *Nicator vireo*

B) *Camaroptera brevicaudata*

C) *Cisticola juncidis*

**Figure S2.** Heterozygosity and sequencing depth (genome coverage) in males (homogametic; ZZ) and females (heterogametic; ZW) across *Parus major* chromosomes (numbered accordingly) in 1 Mb windows for (A) *Nicator vireo*, (B) *Camaroptera brevicaudatus* and (C) *Cisticola juncidis*. Sequencing depth is based on data of three different read mismatch criteria (see main text for details).
